# Colorimetric sensing of hydrogen peroxide using capped *Morus nigra*-sawdust deposited zinc oxide nanoparticles via *Trigonella foenum* extract

**DOI:** 10.3389/fbioe.2024.1338920

**Published:** 2024-02-08

**Authors:** Umar Nishan, Tabassum Zahra, Amir Badshah, Nawshad Muhammad, Saifullah Afridi, Mohibullah Shah, Naeem Khan, Muhammad Asad, Riaz Ullah, Essam A. Ali, Ke Chen

**Affiliations:** ^1^ Department of Chemistry, Kohat University of Science and Technology, Kohat, Pakistan; ^2^ Department of Dental Materials, Institute of Basic Medical Sciences Khyber Medical University, Peshawar, Pakistan; ^3^ Department of Biochemistry, Bahauddin Zakariya University, Multan, Pakistan; ^4^ Department of Pharmacognosy, College of Pharmacy, King Saud University, Riyadh, Saudi Arabia; ^5^ Department of Pharmaceutical Chemistry, College of Pharmacy, King Saud University Riyadh, Riyadh, Saudi Arabia; ^6^ Department of Infectious Diseases, The Affiliated Hospital of Southwest Medical University, Luzhou, China

**Keywords:** diabetes, cancer, TMB, hydrothermal process, colorimetric biosensing

## Abstract

Hydrogen peroxide (H_2_O_2_) is one of the main byproducts of most enzymatic reactions, and its detection is very important in disease conditions. Due to its essential role in healthcare, the food industry, and environmental research, accurate H_2_O_2_ determination is a prerequisite. In the present work, Morus nigra *sawdust deposited* zinc oxide (ZnO) nanoparticles (NPs) were synthesized by the use of Trigonella foenum extract via a hydrothermal process. The synthesized platform was characterized by various techniques, including UV-Vis, FTIR, XRD, SEM, EDX, etc. FTIR confirmed the presence of a Zn‒O characteristic peak, and XRD showed the hexagonal phase of ZnO NPs with a 35 nm particle size. The EDX analysis confirmed the presence of Zn and O. SEM images showed that the as-prepared nanoparticles are distributed uniformly on the surface of sawdust. The proposed platform (acetic acid-capped ZnO NPs deposited sawdust) functions as a mimic enzyme for the detection of H_2_O_2_ in the presence of 3,3′,5,5′-tetramethylbenzidine (TMB) colorimetrically. To get the best results, many key parameters, such as the amount of sawdust-deposited nanoparticles, TMB concentration, pH, and incubation time were optimized. With a linear range of 0.001–0.360 μM and an R^2^ value of 0.999, the proposed biosensor’s 0.81 nM limit of quantification (LOQ) and 0.24 nM limit of detection (LOD) were predicted, respectively. The best response for the proposed biosensor was observed at pH 7, room temperature, and 5 min of incubation time. The acetic acid-capped sawdust deposited ZnO NPs biosensor was also used to detect H_2_O_2_ in blood serum samples of diabetic patients and suggest a suitable candidate for *in vitro* diagnostics and commercial purposes.

## 1 Introduction

Hydrogen peroxide (H_2_O_2_) monitoring in diverse matrices has crucial roles in cell metabolism and has diverse applications in industrial processes (Zhang et al., 2018). H_2_O_2_ is used in medical diagnostics, clinical research and industrial sectors including textiles, paper, pharmaceuticals, food processing, cleaning, disinfection, etc (Patel et al., 2020). Additionally, in the biosystem, it regulates metabolic activity, cell apoptosis, immune cell activation, and different physiological processes (Miller et al., 2010). It serves as an oxidative agent, a stress marker, and a cell defensive agent. Similarly, it is an important biomarker for a variety of diseases and disorders, including cardiovascular, Alzheimer’s, Parkinson’s, diabetes, and neurodegenerative disorders (Rao and Balachandran, 2002). Furthermore, H_2_O_2_ is a byproduct of lactate, alcohol, glucose, glutamate, and cholesterol oxidases. In the past, different detection methods for H_2_O_2_ quantifications were applied. These include chemiluminescence (Irani-nezhad et al., 2019), chromatography (Nakashima et al., 1994), electrochemistry (Lee et al., 2016), fluorescence (Senthamizhan et al., 2016), electrochemical methods, etc (Chen et al., 2014). However, most of these approaches are toxic to living cells, thus making them ineffective for *in situ* H_2_O_2_ detection in biological materials. Besides, some of these techniques are time-consuming, expensive, and complex, restricting their application in laboratories with limited resources (Nishan et al., 2021a). Conversely, in comparison to other complex approaches, colorimetric methods for detecting H_2_O_2_ have been getting key attention nowadays due to their easy handling and low cost. The progress of colorimetric reactions can be monitored with the naked eye (Khaliq et al., 2023).

Cellulose is the main constituent of sawdust. It is one of the most abundant, natural, renewable, biocompatible, and environmentally friendly macromolecules (Park et al., 2019). Cellulosic materials have adaptable surface characteristics, low cost, better mechanical properties, a higher aspect ratio, a lower density, a higher surface area, and a lower density. Cellulose-based sawdust has been utilized as a sacrificial porous template because it is non-edible, cheap, renewable, and readily available biomass (Khaliq et al., 2023).

Various nanomaterials, including positively charged gold nanoparticles (Jv et al., 2010), CuS nanoparticles (Dutta et al., 2013), graphene oxide (Song et al., 2010), ceria nanoparticles (Ornatska et al., 2011), cupric oxide nanoparticles (Chen et al., 2011), and CoFe_2_O_4_ NPs (Shi et al., 2011), have been found to exhibit peroxidase-like activity and are employed to detect H_2_O_2_ visually. Additionally, acetic acid-capped ZnO NPs are recyclable, highly stable, and efficient, have good sensing and catalytic capabilities, and have a tremendous potential to replace expensive noble metal NPs in biosensing. Because ZnO NPs have a large band gap (3.3 eV), they can be employed for UV luminescence at room temperature (Khranovskyy et al., 2012). Furthermore, ZnO NPs have a high isoelectric point (pI) of 9.5, allowing effective immobilization of enzymes with a low pI, i.e., ≤5 (Wei et al., 2010). ZnO NPs are also biocompatible, have the largest family of nanostructures, are crystalline, and have a high surface-to-volume ratio (Abou Chaaya et al., 2014).

In the present study, the hydrothermal method was used for the synthesis of *Morus nigra-*deposited ZnO NPs with the use of *Trigonella foenum* extract as a reductant. To further improve their sensing abilities, the synthesized NPs were capped with acetic acid. The oxidation of chromogenic substrate, i.e., TMB, by H_2_O_2_ in the presence of acetic acid-capped sawdust deposited@ZnO is being reported for the first time. The proposed platform is a new, simple, quick, highly sensitive, and selective approach for H_2_O_2_ detection. The amount of capped NPs, pH, TMB concentration, and incubation time were among the various reaction parameters that were adjusted to achieve the best performance out of the suggested sensor. The sensitivity and selectivity were also investigated under the aforementioned optimum conditions. Finally, H_2_O_2_ levels were also measured in blood serum samples to testify to the fabricated platform.

## 2 Experimental procedure

### 2.1 Materials and reagents

In the entire experimental procedure, all chemicals used were of analytical grade, and no further purification was performed. Double-distilled water was used in the preparation of solutions. NaOH (97%), HCl (37%), acetic acid (97%), ascorbic acid (97%), urea (99.5%), and 3,3′,5,5′-tetramethylbenzidine (TMB) were procured from Daejung, South Korea. KGaA and H_2_O_2_ (35%) were purchased from Merck. The collection of blood serum was performed at a local lab close to the divisional teaching hospital in Kohat, KP, from three diabetic individuals. The serum was twice diluted with a PBS solution to decrease the complexity of the matrix, according to the earlier reports (Singh et al., 2022).

### 2.2 Instrumentation

Fourier transform infrared spectroscopy (FTIR, Nicolet 6,700, US) was used to find the characteristic peaks of the synthesized platform in the range of 4,000–500 cm^-1^. The morphology of the synthesized NPs was confirmed using a scanning electron microscope (SEM) with INCAx-act Oxford Instruments (TESCAN VEGA (LMU)). X-ray diffraction was used to identify the phases of the produced ZnO NPs (JCPDS, file No. 04-0783). The absorption spectra were taken with a Shimadzu UV-Vis spectrophotometer (1,800, Japan).

### 2.3 Synthesis of Sawdust-deposited@ZnO NPs

The green leaves of *T. foenum* were collected, washed with distilled water, and dried in sunlight for 4 days. The leaves were ground into a fine powder with the help of a blender. Extract was prepared in distilled water by suspending 5 g of leaf powder in 200 mL of distilled water on a hot plate with a magnetic stirrer (1,000 rpm) for 1 h at 65°C. The mixture was filtered, and the extract was poured into a beaker. One Gram of zinc acetate was dissolved in 50 mL of distilled water and placed on a hot plate. Subsequently, 50 mL of extract solution was added dropwise to the zinc acetate solution, and 1 g of sawdust from *M. nigra* was gradually added while stirring at 65°C for 4 h. The synthesized sawdust-deposited@ZnO NPs solution was centrifuged for 15 min at 4,500 rpm to obtain solid material.

### 2.4 Capping of sawdust-deposited@ZnO NPs with acetic acid

The Sawdust-deposited@ZnO NPs were capped with an acetic acid solution such that 0.12 g of the mimic enzyme was mixed with 2 mL of acetic acid for 30 min through a mortar and pestle thoroughly. It resulted in the formation of a brown mixture that was placed in an Eppendorf tube for further use (Asad et al., 2022; Nishan et al., 2022).

### 2.5 Colorimetric sensing of H_2_O_2_

Capped sawdust-deposited@ZnO NPs (25 μL) were suspended in 500 μL phosphate buffer (pH 7), followed by the addition of 150 μL TMB solution (18 mM). Add 90 μL of H_2_O_2_ (0.360 μM) to the reaction mixture and incubate at room temperature for the colorimetric reaction. The absorption spectrum of the resultant solution was recorded using a UV-Vis spectrophotometer. Some experimental parameters, such as response time, pH, the amount of capped NPs, and the concentration of TMB solution, have been tuned up to achieve the best results of the proposed platform.

## 3 Results and discussion

### 3.1 Characterization of the sawdust-deposited@ZnO NPs

#### 3.1.1 UV-vis spectroscopy

**FIGURE 1:**
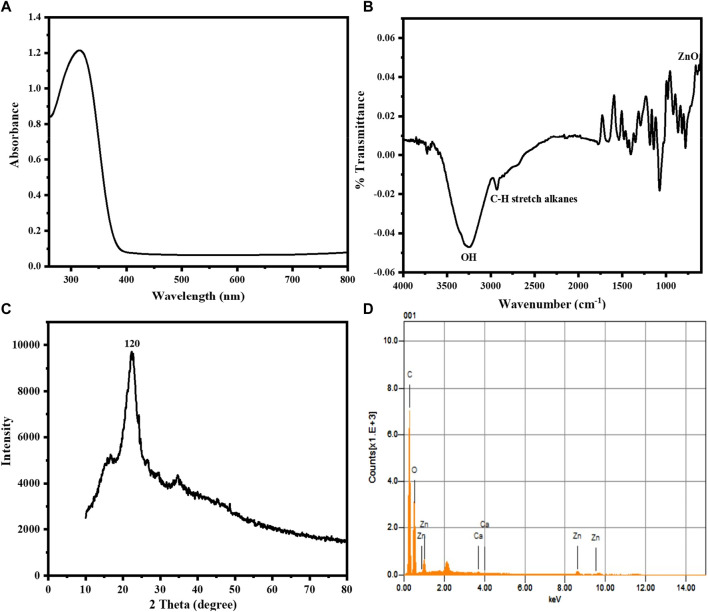
**(A)** UV-Vis absorption spectrum of the sawdust-deposited@ZnO NPs showing its characteristic surface Plasmon resonance peak at 320 nm. **(B)** FTIR spectrum of the synthesized sawdust-deposited@ZnO NPs showing the presence of a ZnO band. **(C)** XRD pattern of the synthesized sawdust-deposited@ZnO NPs, indicating the orthorhombic phase of ZnO. **(D)** EDX spectral analysis of the synthesized sawdust-deposited@ZnO showing the presence of C, Zn, and O.

To investigate the optical characteristics of the sawdust-deposited@ZnO NPs, a UV-Visible spectrophotometer was used. The UV-Vis absorption spectrum of the synthesized sawdust-deposited@ZnO NPs with a peculiar absorption band at 320 nm is shown in Figure 1A. The fact that ZnO has a considerable, sharp absorption implies that the nanoparticles distribution is monodispersed (Talam et al., 2012).

#### 3.1.2 FTIR analysis of the sawdust-deposited@ZnO NPs

FTIR analysis in the range of 4,000–500 cm^-1^, was used to determine the different functional groups found on the surface of the sawdust-deposited@ZnO NPs. The broad absorption band at 3,310 cm^-1^ indicates the presence of an OH group from the plant source on the surface of the synthesized platform. The peak around 2,950 cm^-1^ shows the C-H stretching vibration of the alkyl group present in the mimic enzyme. The most important characteristic peak around 580 cm^-1^ represents the presence of Zn‒O bond present in our synthesized platform, indicating that the ZnO nanoparticles present in the sawdust-deposited@ZnO NPs are as shown in Figure 1B. A similar pattern of peaks has already been reported for ZnO in the literature (Xiong et al., 2006).

#### 3.1.3 XRD analysis

The X-ray diffraction pattern of the synthesized sawdust-deposited@ZnO NPs is shown in Figure 1C. The XRD results of the prepared platform centered at 2θ = 21 reveal a diffraction peak with miller indices of 120. When compared to standard data, it was found that the peak matched the hexagonal phase of ZnO NPs standard data (JCPDS card no. 36–1451) (Srivastava et al., 2013). The average crystal size of orthorhombic-phase ZnO NPs was calculated to be about 35 nm using Scherer equation.

#### 3.1.4 EDX analysis

The chemical composition of the sawdust-deposited@ZnO NPs was examined using EDX analysis, as shown in Figure 1D and Table 1. The results showed the presence of Zn and O in the sawdust-deposited@ZnO sample. In addition to Zn and O, some other elements like Ca and C are also present. The percent contents of Zn, O, C, and Ca are 3.64, 46.95, 49.90, and 0.17, respectively, by weight as shown in the table.

**TABLE 1:** EDX elemental analysis of the synthesized sawdust-deposited@ZnO

Element	Weight %	Atomic %
C	49.90	58.45
O	46.95	40.71
Ca	0.17	0.06
Zn	3.64	0.78
Total	100.00	100.00

#### 3.1.5 SEM analysis

**FIGURE 2:**
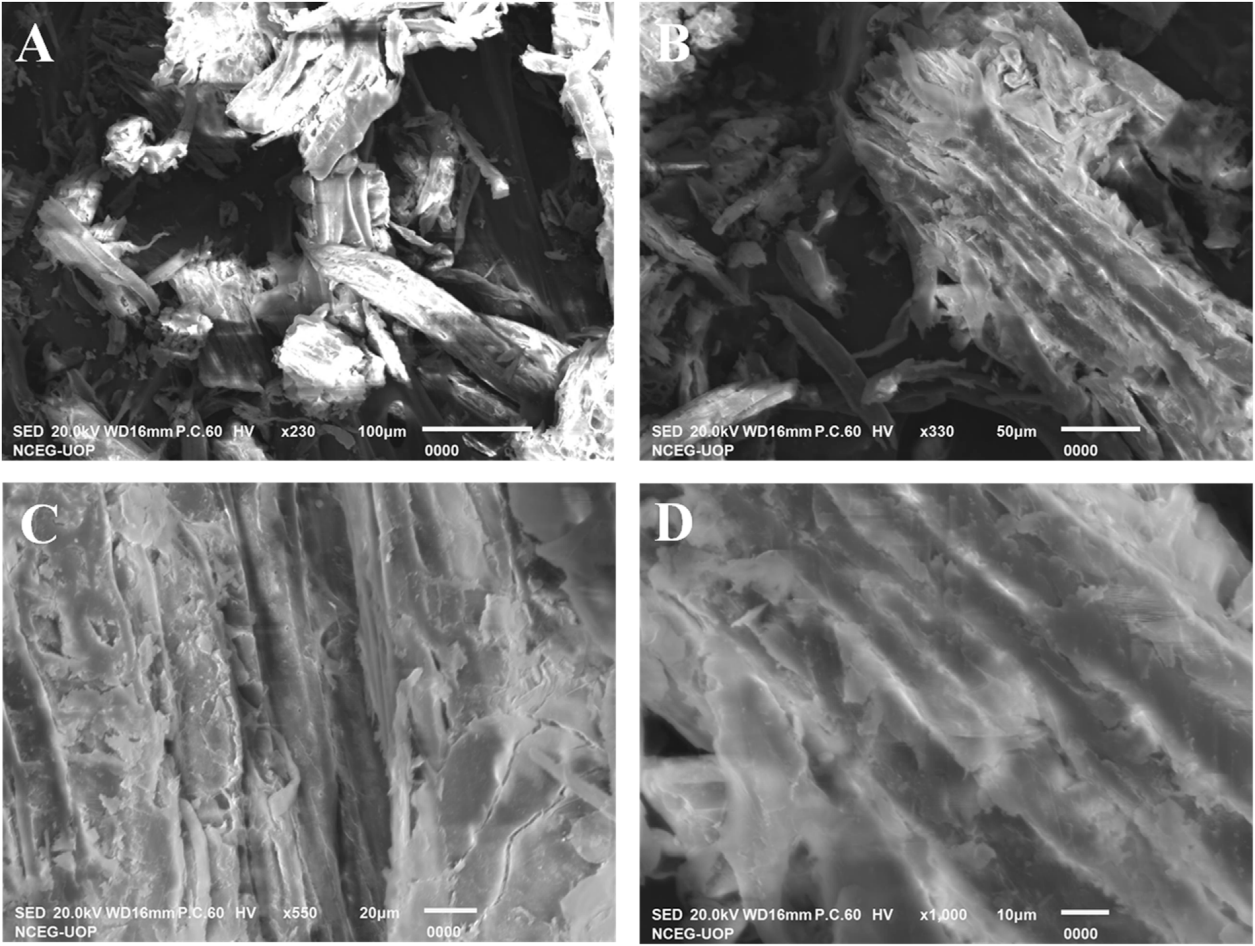
SEM images **(A‒D)** were taken at different magnifications. The results depict that the nanoparticles are distributed uniformly over the surface of sawdust to enhance its catalytic activity.

To investigate the surface morphology of the synthesized sawdust-deposited@ZnO NPs, SEM images of different resolutions were taken, as shown in Figure 2A−D. SEM images confirmed that the prepared ZnO NPs are distributed uniformly over the surface of sawdust. This uniform distribution of the nanoparticles is highly desirable and helpful in terms of the surface area of the nanoparticles for their catalytic activity.

### 3.2 Colorimetric detection of H_2_O_2_

**FIGURE 3:**
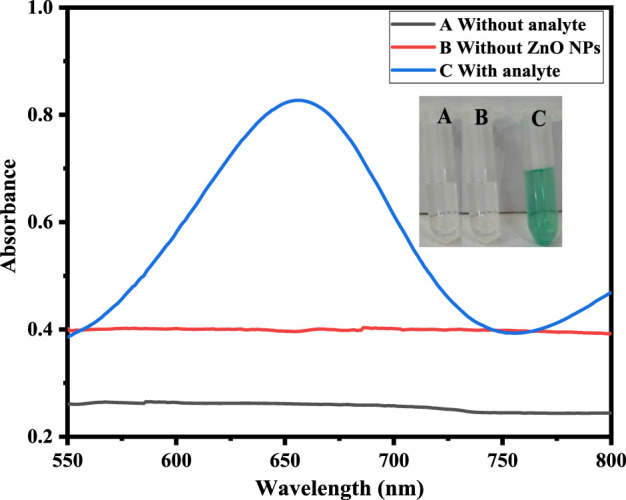
Shows sawdust-deposited@ZnO NPs-based H_2_O_2_ biosensing. It shows UV-Vis spectra of the solution containing capped sawdust-deposited@ZnO NPs (20 μL), PBS pH 7 (500 μL), TMB (18 mM: 150 µL), and H_2_O_2_ (0.360 μM: 100 µL). Spectra were obtained without H_2_O_2_ as well as in the presence of H_2_O_2_. The Figure shows the UV-Vis spectra of a peculiar solution. Curve A represents the reaction system without analyte, B represents the reaction in which sawdust was used without ZnO NPs. Curve C represents the colorimetric change and peak at 652 nm that occurred when H_2_O_2_ was introduced to the sawdust-deposited@ZnO NPs.

H_2_O_2_ sensing by the proposed sawdust-deposited@ZnO NPs was done using a very simple and highly selective colorimetric approach. The optical sensing and UV-Vis absorption spectra are shown in Figure 3. When H_2_O_2_ is introduced to the sensor system, it produces a blue-green color from the colorless TMB. Mechanistically, adsorption of H_2_O_2_ on the surface of NPs produces OH radicals, which oxidize the colorless TMB substrate into a blue-green product, as can be seen with the naked eye, as shown in Figure. The colorimetric change was confirmed by a UV-Vis spectrophotometer. To confirm that the colorimetric change was due to the synthesized sawdust-deposited@ZnO NPs, we used *M. nigra* sawdust without ZnO NPs as a negative control. When H_2_O_2_ was added, no color change was detected; indicating that the color change was caused only by the capped sawdust-deposited@ZnO NPs. UV-Vis spectroscopic investigation validated the negative control experiment, as indicated in Figure.

### 3.3 Proposed mechanism of the reaction

**SCHEME 1:**
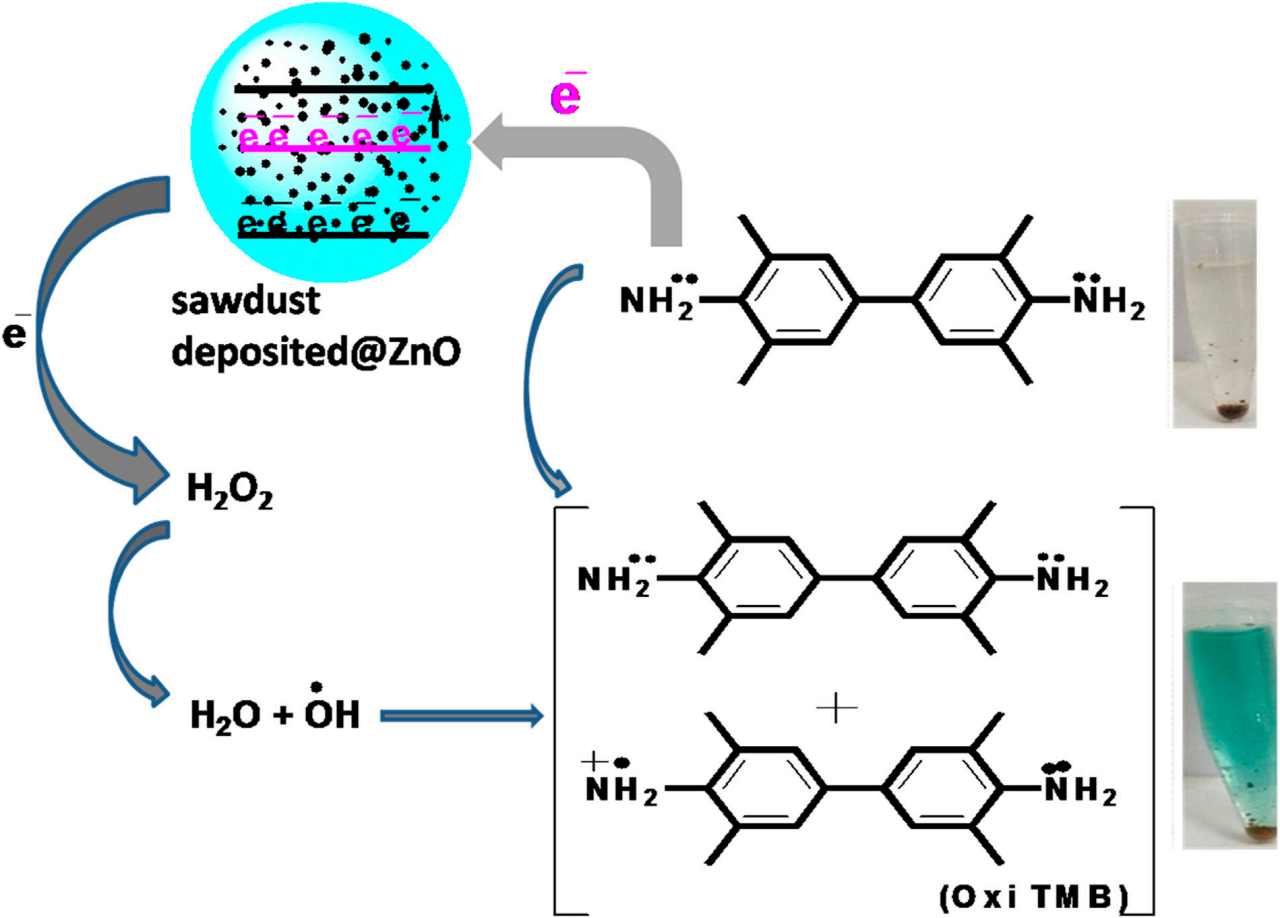
Showing the proposed reaction for the colorimetric sensing of hydrogen peroxide based on the fabricated mimic enzyme.

In the current work, mimic enzyme (acetic acid-capped ZnO NPs deposited sawdust) receive electrons from TMB. It results in an increase in the conductivity of electrons in the mimic enzyme, which provides an active site for the proposed chemical reaction. The mobility of electrons results in the transfer of electrons to H_2_O_2_. It results in the generation of hydroxyl free radicals. The generated hydroxyl free radicals oxidize the TMB, resulting in the formation of a blue-green complex. This colorimetric change is visible to the naked eye and was also confirmed with a UV-Vis spectrophotometer. The maximum absorption was found to be at 652 nm. The detailed proposed reaction can be in seen in Scheme 1.

### 3.4 Optimization of parameters

#### 3.4.1 Amount of capped ZnO NPs

**FIGURE 4:**
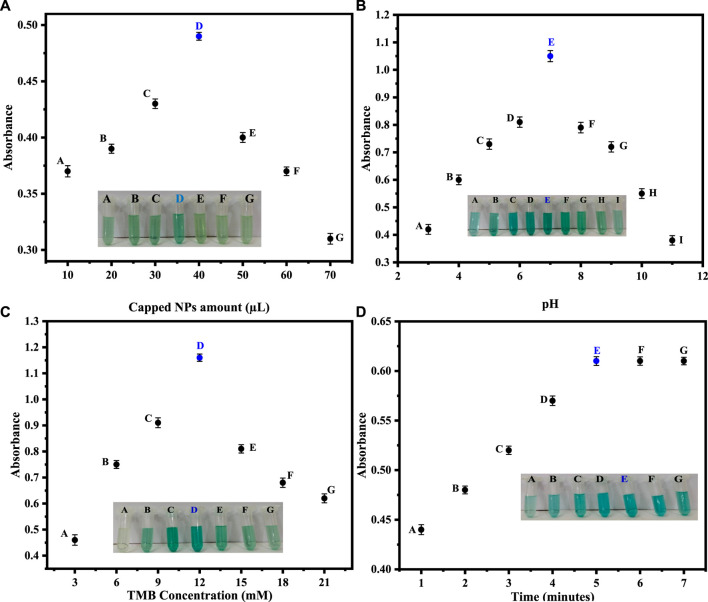
**(A)** Optimization of the capped sawdust-deposited@ZnO NPs [Reaction cond. PBS 500 µL (pH 7), TMB 150 µL (18 mM), H_2_O_2_ 100 µL (0.360 μM). **(B)** Different pH optimizations for the proposed capped sawdust-deposited@ZnO NPs show the best colorimetric response at pH 7. [Reaction cond. Capping 20 μL, TMB 150 µL (18 mM), H_2_O_2_ 100 µL (0.360 μM)]. **(C)** The optimization of TMB concentration of the capped sawdust-deposited@ZnO NPs [Reaction cond. Capping 40 μL, PBS 500 µL (pH 7), H_2_O_2_ 100 µL (0.360 μM)]. **(D)** Reaction time optimization for the suggested capped sawdust-deposited@ZnO NPs [reaction cond. Capping 40 μL, PBS 500 µL (pH 7), TMB 150 µL (12 mM), H_2_O_2_ 100 µL (0.360 μM)].

In order to get the best colorimetric response, we first optimized the amount of capped sawdust-deposited@ZnO NPs. Briefly, different amounts (10–70 µL) of the capped sawdust-deposited@ZnO NPs were tested, and the best colorimetric response was obtained at a 40 µL concentration, as shown in Figure 4A. No significant colorimetric response was obtained below 40 μL, so the 40 µL amount was taken as the optimum amount for further experiments. Previously, we reported about 25 μL of capped TiO_2_ NPs as an optimum concentration for the colorimetric sensing of H_2_O_2_ (Nishan et al., 2021a). Under the given conditions, an increase in the concentration of the mimic enzyme from 40 µL up to 70 µL results in a lower response. This can possibly be explained by the fact that unreacted mimic enzyme interferes with the already oxidized TMB, resulting in much lower absorption.

#### 3.4.2 pH optimization

Different pH optimizations were done to get the maximum colorimetric response. Briefly, different pH solutions of PBS were made, and their respective pH values were adjusted using sodium hydroxide and hydrochloric acid solutions. The best colorimetric response shown by the capped sawdust-deposited@ZnO NPs was recorded on pH 7, as shown in Figure 4B. No significant colorimetric response was noticed above or below this optimum pH of 7, therefore pH 7 was selected as the optimum pH for further experiments. At a lower pH, the concentration of hydrogen ions increases, which results in the protonation of the amino group of the chromogenic substrate TMB. This protonation of TMB makes it less susceptible to oxidation, resulting in a lower colorimetric change. The increase in pH above 7 results in an increase in hydroxyl ion concentration. As a result, the oxidation of TMB reduces, and hence less colorimetric change can be observed. Similarly an earlier study reported pH 7.5 to be optimum for the colorimetric sensing of hydrogen peroxide (Nishan et al., 2021b).

#### 3.4.3 Optimization of TMB concentration

TMB solutions of different concentrations ranging from 3 to 21 mM were prepared. Initially, the colorimetric response increased up to 12 mM and then decreased as the concentration of TMB increased from 13 mM to 21 mM. The best colorimetric response was noticed at 12 mM, as shown in Figure 4C. Recently, for the detection of hydrogen peroxide, the 8 mM optimum TMB concentration was reported by our groups for another nanostructure (Nishan et al., 2023). This could possibly be explained by the fact that in the reported work, a pristine form of nanomaterial functionalized with ionic liquid was used as a mimic enzyme. In the current work, sawdust was used as a matrix material, hence the higher concentration of TMB.

#### 3.4.4 Optimization of time

In colorimetric detection of hydrogen peroxide, the reaction incubation time was also optimized. A colorimetric response was noticed at various time intervals (1–7 min) after adding hydrogen peroxide. The reaction time at various intervals was recorded by UV-Vis spectroscopy. After 5 min, no further change in color or absorbance was noticed, indicating that 5 min is the optimal time for a complete reaction, as shown in Figure 4D. According to the literature, the optimum time for the detection of hydrogen peroxide was 10 min, as reported by (Zarif et al., 2020), which is much higher than our present work.

### 3.5 Optimization of hydrogen peroxide concentration

**FIGURE 5:**
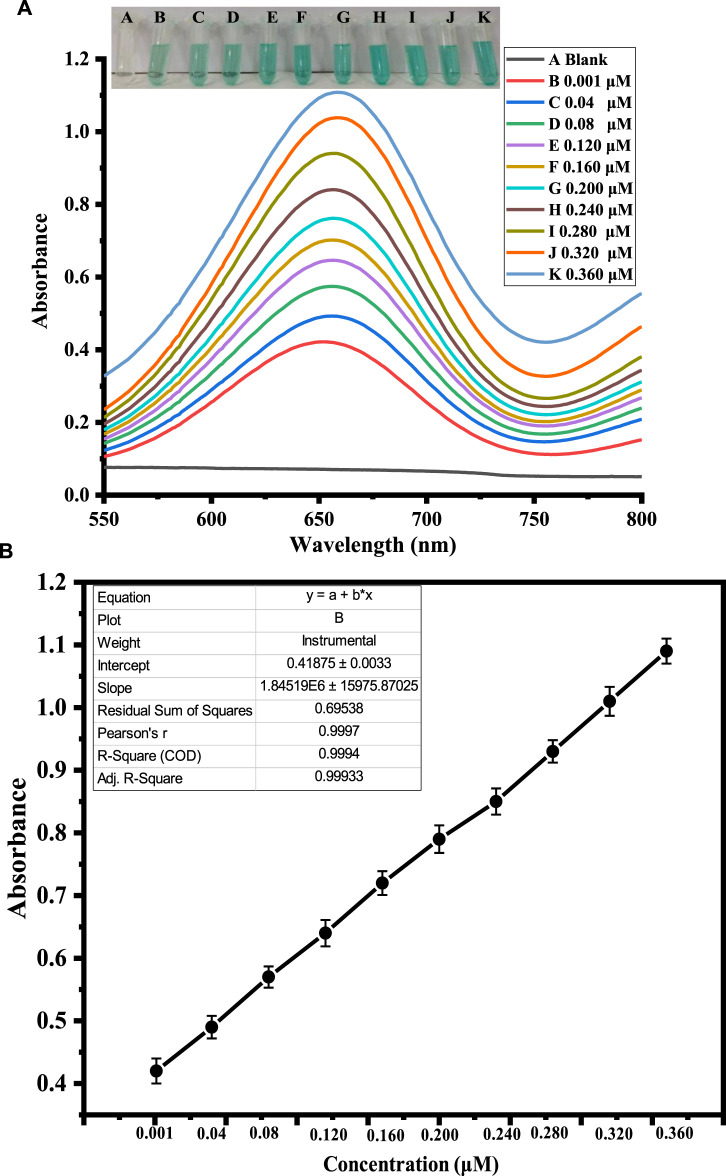
Shows the optimization of H_2_O_2_ concentration. Figure **(A)** shows the UV-Vis response recorded at different concentrations of H_2_O_2._ The inset figure shows varying color changes with the addition of different concentrations of H_2_O_2_. Figure **(B)** shows the corresponding calibration curve of the absorbance at different H_2_O_2_ concentrations.

H_2_O_2_ was detected using a quick and easy colorimetric method based on capped sawdust-deposited@ZnO NPs under ideal experimental circumstances. As seen in Figure 5, the developed biosensor’s sensitivity for H_2_O_2_ detection was tested using a range of H_2_O_2_ concentrations. At lower H_2_O_2_ concentrations, the sensor response and peak intensity were negligible, but as the concentration rose, they grew linearly. H_2_O_2_ detection with an R^2^ value of 0.999 and a linear range of 0.001–0.360 μM was made possible by this method. It was determined that the limits of quantification (LOQ) and detection (LOD) were, respectively, 0.24 nM and 0.81 nM. The suggested colorimetric approach had the advantages of a low detection limit, low cost, and naked eye observation over other previously published detection methods. Based on the linear range and limit of detection, we compared this work for H_2_O_2_ detection with previously reported colorimetric approaches, as shown in Table 2. It is clear from the results that the fabricated sensor showed an exceptional limit of detection and a comparable wide linear range with previous works from our group.

**TABLE 2:** Comparison of different colorimetric biosensors for H_2_O_2_.

S. No.	Materials used	Method applied	Linear range (μM)	Limit of detection (μM)	References
1	CuS	Colorimetric	1–1,000	0.11	Guan et al. (2015)
2	PB NPs	Colorimetric	0.1–50	0.031	Zhang et al. (2014)
3	Ag NPs	Colorimetric	0.01–30	0.014	Teodoro et al. (2019)
4	RhNPs	Colorimetric	1–100	0.75	Choleva et al. (2018)
5	Cu(II)-coated Fe_3_O_4_ NPs	Colorimetric	2.5–100	0.2	Liu et al. (2019)
6	GQDs/CuO	Colorimetric	0.5–10	0.17	Zhang et al. (2017)
7	ZV-Mn NPs	Colorimetric	10–280	0.2	Rauf et al. (2020)
8	FeCDs	Colorimetric	6–42	0.93	Bandi et al. (2021)
9	Ni NPs	Colorimetric	400–4,000	120	Zarif et al. (2020)
10	TiO2 NPs	Colorimetric	0.001–0.360	0.08	Nishan et al. (2021a)
11	lignin-based Ag NPs	Colorimetric	0.001–0.360	0.0137	Nishan et al. (2021b)
12	Ag-Fe2O3 NPs	Colorimetric	0.001–0.320	0.0107	Nishan et al. (2023)
13	Capped sawdust-deposited@ZnO NPs	Colorimetric	0.001–0.360	0.00024	This work

### 3.6 Selectivity analysis the proposed sensor

**FIGURE 6:**
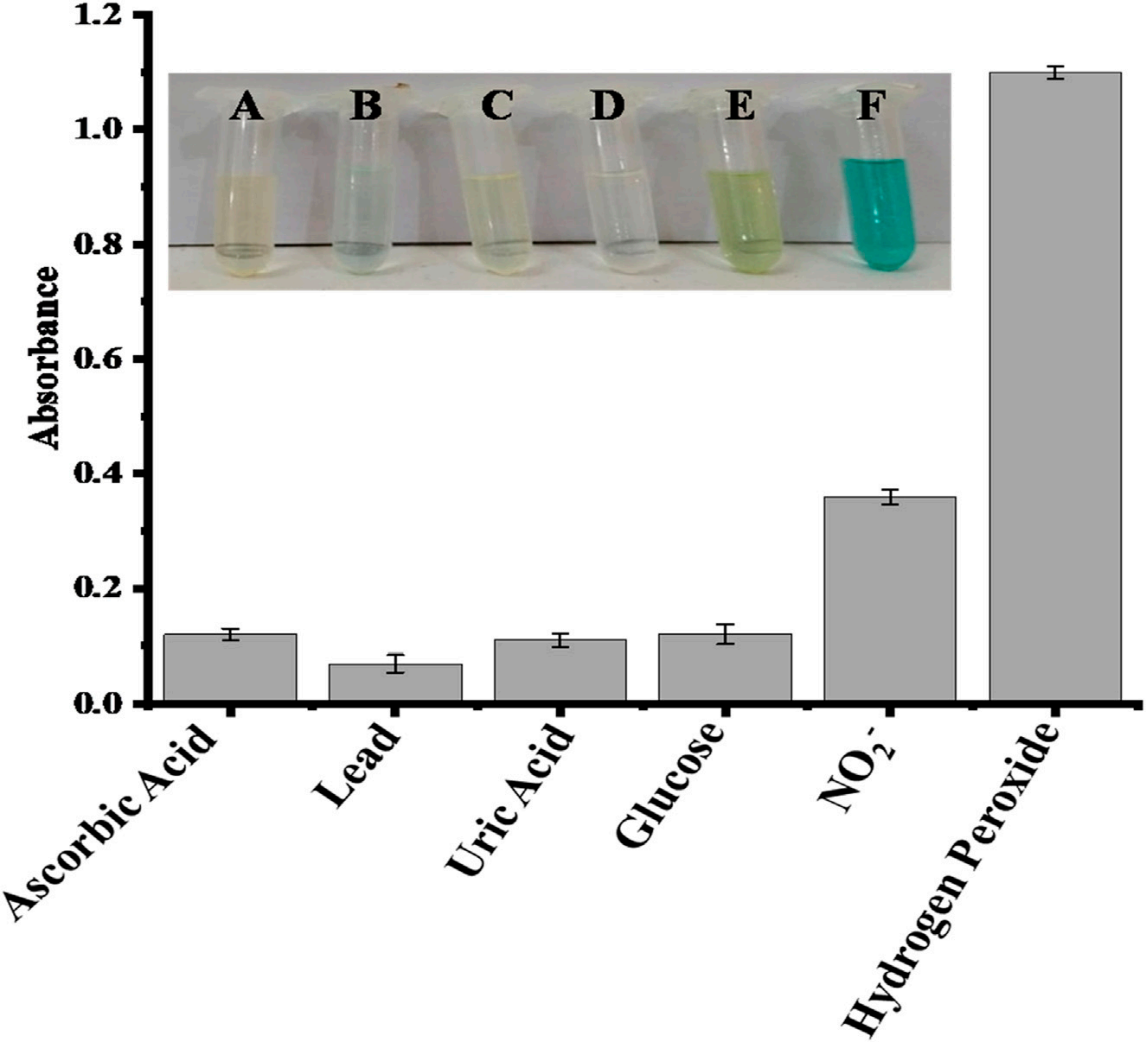
Comparative interference study of the proposed capped sawdust-deposited@ZnO NPs sensor for the detection of hydrogen peroxide with other analytes, indicating high selectivity of the proposed platform for the sensing of H_2_O_2_. In the inset Figure, the letters A, B, C, D, E, and F represent ascorbic acid, lead, uric acid, glucose, nitrite, and hydrogen peroxide, respectively.

The potential interfering chemicals, including ascorbic acid, lead, uric acid, glucose, and nitrite, were used to test the selectivity of the proposed sensor. All these interfering chemicals had substantially lower absorbance than H_2_O_2_, as shown in Figure 6. The recorded absorbance was highest when H_2_O_2_ was added, and no significant absorbance change was seen when a co-existing material was added. In the presence of higher amounts of ascorbic acid, lead, uric acid, glucose, and nitrite ions, the suggested sensor has a substantially stronger selectivity for H_2_O_2_. All the experiments were performed in the presence of 0.360 μM H_2_O_2_ and a double concentration of other interfering substances.

### 3.7 Real sample analysis

**FIGURE 7:**
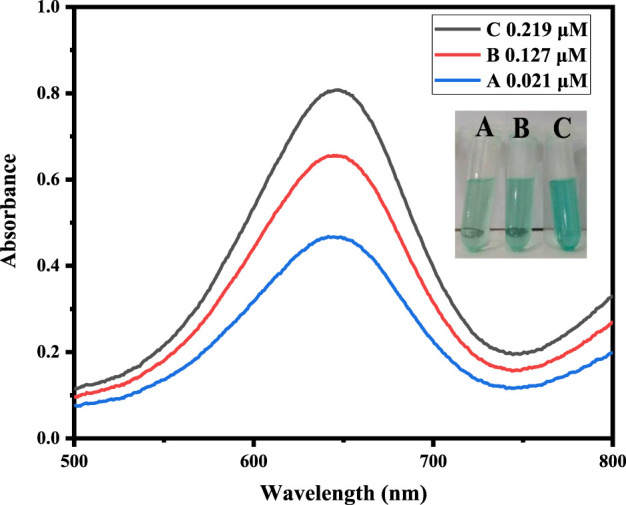
Real sample analysis of a blood serum sample of a diabetes patient at optimized conditions by the addition of different concentrations of hydrogen peroxide, such as 0.017, 0.120, and 0.206 μM.

To assess the practical application of the proposed sensor to detect H_2_O_2_ content, the measurement of H_2_O_2_ in the blood serum sample of a diabetes patient was carried out as shown in Table 3. The present amount of H_2_O_2_ was calculated from the already calibrated graph by using the spiking method. Different concentrations of H_2_O_2_ solution, such as 0.017, 0.120, and 0.206 μM, were spiked into the blood serum sample of a diabetes patient and analyzed, as shown in Figure 7. The results demonstrated that the H_2_O_2_ concentrations in the real samples determined by the current assay are in good agreement with the spiked H_2_O_2_ concentrations.

**TABLE 3:** Detection of hydrogen peroxide in blood serum sample of diabetes patient (n = 3).

Samples	Detected (μM)	H_2_O_2_ added (μM)	H_2_O_2_ found (μM)	Recovery (%)	RSD (%)
1	0.004	0.017	0.021	123.53	0.271
2	0.007	0.120	0.127	105.83	0.451
3	0.013	0.206	0.219	106.31	0.214

## 4 Conclusion

*Morus nigra*-deposited ZnO@NPs were successfully synthesized from *T. foenum* extract. The synthesized platform was characterized with various standard analytical techniques, including FTIR, SEM, XRD, and EDX. The synthesized sawdust-deposited@ZnO NPs were capped with acetic acid and successfully used for the colorimetric sensing of H_2_O_2_. Our current finding demonstrates that acetic acid-capped sawdust-deposited@ZnO NPs show enhanced intrinsic peroxidase-like activity. The proposed platform showed good sensitivity and selectivity in the presence of a double amount of potential interfering species. The fabricated platform shows a number of advantages over natural enzymes, including easy preparation, low cost, quick reaction times, and high stability. These advantages make it a suitable candidate peroxidase-mimic for future applications in biotechnology, medical diagnostics, and hydrogen peroxide monitoring.

## Data Availability

The raw data supporting the conclusion of this article will be made available by the authors, without undue reservation.
